# The Clinical Impact of SARS-CoV-2 on Hypertrophic Cardiomyopathy

**DOI:** 10.3390/jcdd11040104

**Published:** 2024-03-29

**Authors:** Danish Saleh, Zhiying Meng, Nicholas Johnson, Abigail Baldridge, Allison R. Zielinski, Lubna Choudhury

**Affiliations:** 1 Division of Cardiology, Department of Medicine, Northwestern University, Feinberg School of Medicine, Chicago, IL 60611, USA; 2 Bluhm Cardiovascular Institute Clinical Trials Unit (BCVI-CTU), Northwestern University, Feinberg School of Medicine, Chicago, IL 60611, USA; 3 Information Technology, Research Analytics, Northwestern University, Feinberg School of Medicine, Chicago, IL 60611, USA; 4 Bluhm Cardiovascular Institute, Northwestern University, Feinberg School of Medicine, Chicago, IL 60611, USA

**Keywords:** Hypertrophic Cardiomyopathy, SARS-CoV-2, COVID-19, heart failure, myocardial injury, mortality

## Abstract

Background: This study aims to understand and describe the clinical impact of SARS-CoV-2 (COVID-19) infection in patients with Hypertrophic Cardiomyopathy (HCM). Methods: A data repository of over 6.6 million patients in a large metropolitan (Chicago IL) healthcare system was queried to identify adults with a history of HCM and COVID-19 infection between 2019 and 2021. Propensity score-matched analysis was performed based on age, sex, BMI, and elements of the cardiovascular history, including tobacco use, hypertension, hyperlipidemia, myocardial injury, and heart failure. Results: Individuals with HCM and COVID-19 infection had more total hospitalizations (41.6 v 23 per 100 persons, *p* < 0.01), more heart-failure-related hospitalizations (24.2 v 8.7 per 100-persons, *p* < 0.01), more non-ST elevation myocardial injury (NSTEMI) hospitalizations (8.6 v 4.6 per 100-persons, *p* < 0.01), and increased mortality (10.8 v 5 per 100-persons, *p* < 0.01) compared to HCM patients without a history of COVID-19 infection. Patients with HCM and COVID-19 were also noted to have a higher peak CRP when compared to those without prior COVID-19 (Inter-quartile range of 9.0–106.9 v 1.8–21.3, *p* < 0.01). Conclusions: In patients with HCM, COVID-19 infection is associated with increased incidence of myocardial injury, increased number of total and heart-failure specific hospitalizations, and increased mortality.

## 1. Introduction

Hypertrophic Cardiomyopathy (HCM) is an autosomal dominant genetic disorder that impacts sarcomere formation resulting in myofibril disarray and fibrosis of the heart muscle [1]. Epidemiologic studies estimate that the prevalence of HCM is 1:500 to 1:200 in the general population and affects more than 20-million people worldwide [2,3]. Asymmetric hypertrophy of the myocardium is a feature of phenotypic HCM that can provoke left-ventricular outflow tract obstruction and typical symptoms. Clinically, syncope, arrhythmia (atrial fibrillation, non-sustained ventricular tachycardia, ventricular tachycardia), and/or heart failure are manifestations of advanced disease. Sudden (cardiac) death in young athletes has classically been associated with undiagnosed HCM [1,4].

Myocardial scarring and fibrosis in HCM are believed to result from microvascular disease (small vessel ischemia) that causes cardiomyocyte death [1,5]. Emerging evidence suggests that local and systemic inflammation may be critical to the phenotypic manifestation of HCM [6]. Serum cytokines, including Tumor-Necrosis Factor-Alpha (TNFα), Interleukin-6 (IL-6), Monocyte Chemoattractant Protein (MCP-1), Serum Amyloid P (SAP), C-Reactive Protein (CRP), and Interleukin-1 (IL-1) are elevated in patients with HCM [6]. Inflammation and myocardial fibrosis marked by late-gadolinium enhancement (LGE) on Cardiac MRI is commonly observed in HCM [7,8]. Of note, cytokine levels have been shown to correlate directly with the degree of hypertrophy and LGE [7]. Moreover, pathologic assessment of myectomy samples of patients with left-ventricular outflow tract (LVOT) obstruction in the setting of HCM revealed pronounced immune-cell infiltrates within myocardial tissue [9].

COVID-19 is the clinical syndrome emerging from SARS-CoV-2 infection. The virus has infected over 250 million people worldwide and has been identified as the cause of death of over 5 million individuals [10]. Studies have suggested an increased risk of inflammatory injury and cardiac tissue dysfunction in association with COVID-19 [11,12,13]. The center for disease control (CDC) reported a >15-fold increase in the risk of developing myocarditis for patients with COVID-19 compared to counterparts who did not carry the diagnosis [14]. Myocarditis associated with SARS-CoV-2 infection appears to be male-predominant and is demographically similar to trends in myocarditis observed prior to the emergence of COVID-19 [15].

Susceptibility to SARS-CoV-2 infection has been linked to host expression of the receptor Angiotensin-Converting Enzyme 2 (ACE2) and Transmembrane Serine Protease-2 (TMPRSS2). ACE2 is ubiquitously expressed across multiple organ systems, including the heart (cardiomyocytes) and vascular tissue (pericytes) and has been shown to be upregulated in cardiovascular disease [16]. TMPRESS2 is also expressed on cardiomyocytes, endothelial cells, and pericytes [13]. It remains to be understood whether COVID-19 associated cardiovascular damage is a function of direct or indirect viral injury caused by SARS-CoV-2. Specifically, it is unclear whether viral entry into cardiovascular cells is required to drive dysfunction or whether secondary inflammatory mechanisms lead to tissue and organ dysfunction [13].

Acute viral illnesses and COVID-19 have been linked with clinical deterioration, including increased hospitalization and mortality, among individuals with cardiovascular risk factors, cardiovascular comorbidities, and HCM [11,12,17,18]. A study of individuals with HCM and acute clinical deterioration, defined by the presence of heart failure exacerbation and/or ventricular arrhythmias, found that these individuals were more likely to have histologic evidence of myocarditis with polymerase-chain reaction (PCR)-detected viral nucleic acid in myocardial tissue [19]. Desai et al. published a large population-based study examining data prior to COVID-19 vaccine availability and observed that patients with HCM who were infected with SARS-CoV-2 had nearly four times higher odds of mortality compared to counterparts without prior infection [20]. Herein, we perform a similar large population-based study to assess the clinical impact of COVID-19 on patients with HCM; our study serves to establish the external validity of the prior work and extends the inquiry to also examine the impact of COVID-19 on hospitalizations in patients with HCM.

## 2. Methods

**Figure 1:**
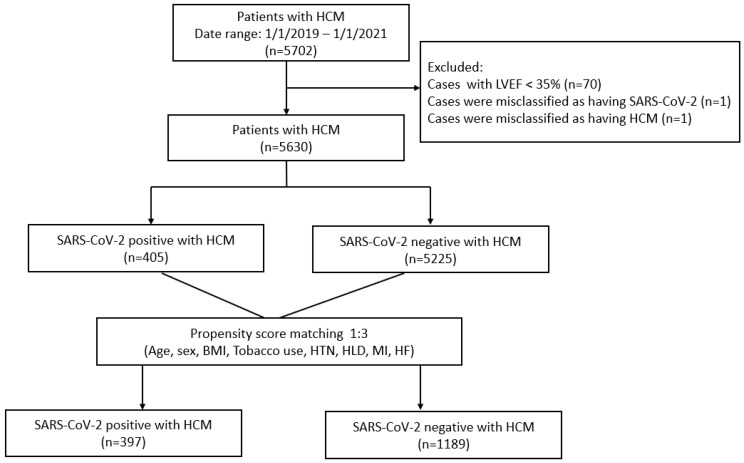
An illustration of the study methodology.

The Northwestern Medicine Enterprise Data Warehouse, a data repository of over 6.6 million patients in a large metropolitan (Chicago, IL, USA) healthcare system was utilized to identify adults aged 18–110 with a history of HCM and/or COVID-19 exposure. Patients were included in the study if COVID-19 infection occurred between January 2019 and January 2021. HCM was determined based on ICD-10 codes corresponding to diagnoses of Hypertrophic Cardiomyopathy (‘I42.1’, ‘I42.2’, ‘425.11’, and ‘425.18’). COVID-19 diagnosis was determined using ICD-10 codes (‘B97.21’, ‘B97.29’, ‘U07.1’, ‘U071.2’, and ‘J12.81’) as well as a review of laboratory testing data within the Northwestern Medicine Healthcare System. Patients with an ejection fraction of lower than 35% were excluded from the study as these are less likely to reflect HCM physiology and are perchance misclassified within the database. The initial query yielded 5702 patients (5284 with HCM without a SARS-CoV-2 positive history and an additional 418 with a history of SARS-CoV-2 infection) eligible for evaluation (Figure 1). Seventy patients with EF < 35% were excluded. A single patient was excluded from the study after having been mis-classified as having SARS-CoV-2. A single patient was excluded from the study after having been mis-classified as having HCM. Data were propensity score-matched (1:3) based on age, sex, BMI, and elements of their cardiovascular history, including tobacco use, hypertension, hyperlipidemia, myocardial injury, and heart failure (Supplemental Table S1). The final analysis included 1189 patients with HCM without prior SARS-CoV-2 infection and 397 patients with HCM and a SARS-CoV-2 positive test or ICD-10 diagnosis of COVID-19. Baseline characteristics and clinical outcomes were compared between HCM patients with and without COVID-19 exposure using Chi-squared, Fisher’s exact test, two-sample t-test, or a Wilcoxon test. Statistical significance was defined as *p* < 0.05. Statistical analysis was performed using SAS (Base 9.4, Cary, NC, USA). Institutional Review Board at Northwestern University was obtained prior to initiation of this study.

## 3. Results

Seven percent of patients with HCM had a record of COVID-19 diagnosis between January 2019 and 2021. We performed a propensity-matched comparison between individuals with a history of HCM and COVID-19 to those without prior COVID-19. The average age of each group was approximately 65 years with a standard deviation of 16 years. Females comprised just over 40% of the study groups. The average BMI of each group was ~30.3 kg/m^2^ with a standard deviation of 6.9. After propensity matching, approximately 6 percent of individuals in each of the study groups were smokers, 80 percent had a history of hypertension, 64% of individuals had hyperlipidemia, ~10% had a history of myocardial injury or infarction, and ~40% had a history of heart failure (Table 1).

During the study period, individuals with HCM and COVID-19 infection had more total hospitalizations (41.6 v 23 per 100 persons, *p* < 0.01), more heart-failure related hospitalizations (24.2 v 8.7 per 100-persons, *p* < 0.01), more non-ST elevation myocardial injury (NSTEMI) hospitalizations (8.6 v 4.6 per 100-persons, *p* < 0.01), and increased mortality (10.8 v 5 per 100-persons, *p* < 0.01) compared to HCM patients without a history of COVID-19 infection. Patients with HCM and COVID-19 were also noted to have a higher peak CRP when compared to those without prior COVID-19 (Inter-quartile range of 9.0–106.9 v 1.8–21.3, *p* < 0.01) (Table 2). Although a trend towards higher peak high-sensitivity Troponin was appreciated for patients with prior COVID-19 exposure when compared to those without prior COVID-19, this was not significant (Inter-quartile range of 8–53 v 9–122, *p* ~ 0.16).

## 4. Discussion

In this study, we examine the impact of acute systemic inflammation as marked by COVID-19 infection on the clinical course of patients with HCM. During the study period, patients with HCM and COVID-19 were observed to have an increased number of total hospitalizations, including an increased number of heart-failure specific and NSTEMI hospitalizations. Notably, COVID-19 infection was associated with increased mortality among patients with HCM. Consistent with the inflammatory nature of COVID-19, patients with HCM and COVID-19 had higher peak inflammatory tone, defined by CRP.

The study interval (1 January 2019 through 1 January 2021) predates the emergence of herd immunity within the population and/or the availability of the COVID-19 vaccines which have been shown to protect against COVID-19-related illness [21,22,23]. Widespread COVID-19 vaccination campaigns in the United States were initiated in January of 2021, and as of 1 February 2023, only 26 million doses of the vaccine had been administered [24]. Accordingly, the study window allowed for the specific evaluation of the impact of COVID-19 and an acute inflammatory event in a susceptible population with known HCM.

Our study finds that 7% of patients with previously diagnosed HCM were diagnosed with COVID-19 between January 2019 and the same date in 2021. The Johns Hopkins Coronavirus Resource center reports that as of 1 January 2021, 20.4 million cases of COVID-19 had been reported in the United States [10]. The US Census Bureau estimated the US national population was 331 million in April of 2020 [25]. These data estimate a 6% COVID-19 infection rate as of January 2021. This observation is similar to the infection rate observed in our study and does not suggest a marked difference in susceptibility to COVID-19 among patients with HCM when compared to the general population.

The observations made here are consistent with previously published studies examining COVID-19 in HCM. Desai et al. reported a 4-fold increase in mortality in patients with HCM and COVID-19 exposure compared to unexposed counterparts [20]. In contrast, we observe only a 2-fold increase in mortality. The smaller effect magnitude may be a function of differences in propensity-score matched analysis performed between the studies. For example, our study performed propensity-matching to include known cardiovascular risk factors (hypertension, hyperlipidemia, and tobacco use) as well as a history of myocardial injury and history of heart failure; similar variables were not adjusted for by Desai et al. 

Two smaller retrospective studies examining COVID-19 in HCM have also been reported. The first study examined 305 patients with HCM and SARS-CoV-2 infection and identified age, NYHA class, LVOT obstruction, and left-ventricular systolic diameter (LVSD) as factors driving mortality risk after multi-variate adjustment [26]. The same study compared mortality of patients with HCM and history of COVID-19 to control patients with history of COVID-19 and without HCM; the authors observed a 2.4-fold increase in mortality and 1.7-fold increase in hospitalization when adjusting for age and sex [26]. The second study described the clinical context of 70 patients with HCM and history of a positive SARS-CoV-2 test from a large urban hospital system in New York between January 2020 and January 2021. Given the relatively small study population, statistical power was insufficient to identify patient-related factors contributing to hospitalization of patients with HCM [27].

The specific mechanisms linking acute infection to decompensation in HCM have been speculated but are not completely understood. It is possible that acute infection may provoke circulatory dehydration (insensible losses and fluid extravasation) thereby exacerbating obstructive physiology in patients with known obstructive HCM. In this case, HCM patients with medically, procedurally, and/or surgically relieved LVOT obstruction might be expected to have improved outcomes [28,29]. Moreover, the specific modality of treating LVOT obstruction may be significant insofar as its ability to reliably achieve resolution of obstructive physiology [28,29]. Conversely, patients with non-obstructive HCM may experience hemodynamic complications related to underlying and/or progressive diastolic dysfunction associated with systemic inflammation. In this scenario, diastolic dysfunction may be exacerbated by subclinical or clinically evident myocardial inflammation [19]. Nevertheless, each of these patient populations are expected to have limited hemodynamic reserve in the setting of established cardiomyopathy.

The retrospective nature of this study is an important limitation which can confound data analytics and interpretation. We have attempted to address this concern by performing a pairwise matched study for key demographic parameters (age, sex, BMI) as well as important elements of the cardiovascular history (tobacco use, hypertension, hyperlipidemia, myocardial injury/infarction, and heart failure). Notably, our findings are consistent with the inflammatory nature of COVID-19 and other reports of COVID-19 in cardiovascular disease, heart failure, and HCM [12,17,18,20,26,27]. Another important limitation of our study arises from the inability to differentiate outcomes for patients with obstructive and non-obstructive HCM. Our data curation methods did not permit generation of a similarly large study examining outcomes for patients with and without outflow tract obstruction; nevertheless, this is an important question that warrants future inquiry.

Collectively, the observations reported here and those previously observed demonstrate that COVID-19 is associated with a more adverse clinical course in the setting of HCM. Of note, COVID-19 infection may be regarded as an example of acute systemic inflammation in the setting of underlying heart disease. Accordingly, these observations likely have prognostic significance in understanding acute-inflammatory injury in patients with HCM. Indeed, infection is a well-described decompensating factor precipitating heart failure [30]. Importantly, these data reaffirm the value of primary and secondary prevention against acute illness in susceptible populations, including those living with HCM. 

## Figures and Tables

**Table 1:** Baseline characteristics for patients with hypertrophic cardiomyopathy comparing SARS-CoV-2 positive versus negative in baseline and propensity score matched cohorts.

Characteristics, no (%)	Unmatched				Propensity Matched ^2^			
	HCM/COVID				HCM/COVID			
		Negative	Positive			Negative	Positive	
	N	(n = 5225)	(n = 405)	*p*-Value ^1^	N	(n = 1189)	(n = 397)	*p*-Value ^1^
Age, mean (SD), years	5630	65.8 ± 16.8	65.0 ± 16.0	0.39	1586	64.4 ± 16.1	65.0 ± 16.1	0.57
Female	5630	2360 (45.2)	170 (42)	0.21	1586	484 (40.7)	168 (42.3)	0.57
BMI, mean (SD)	5372	29.3 ± 6.7	30.2 ± 6.9	0.016	1586	30.3 ± 6.9	30.3 ± 6.9	0.86
**Medical history**								
Active tobacco use	5216	334 (6.9)	23 (5.8)	0.39	1586	68 (5.7)	23 (5.8)	0.96
Hypertension	5630	3402 (65.1)	323 (79.8)	<0.01	1586	942 (79.2)	321 (80.9)	0.48
Hyperlipidemia	5630	2779 (53.2)	255 (63)	<0.01	1586	763 (64.2)	255 (64.2)	0.98
Myocardial injury/infarction	5630	376 (7.2)	41 (10.1)	0.030	1586	126 (10.6)	40 (10.1)	0.77
Myocarditis	5630	7 (0.1)	0 (0)	1.00	1586	1 (0.1)	0 (0)	1.00
Pericarditis	5630	22 (0.4)	4 (1)	0.11	1586	7 (0.6)	4 (1)	0.48
Atrial fibrillation	5630	992 (19)	83 (20.5)	0.46	1586	249 (20.9)	83 (20.9)	0.99
NSVT	5630	661 (12.7)	51 (12.6)	0.97	1586	155 (13)	51 (12.8)	0.92
Heart failure	5630	1956 (37.4)	161 (39.8)	0.35	1586	470 (39.5)	160 (40.3)	0.78

^1^ Chi-squared, Fisher’s exact test, two sample t-test, or Wilcoxon test as appropriate. ^2^ The HCM/COVID positive group was 1:3 matched on age, sex, tobacco use, hypertension, hyperlipidemia, myocardial injury/infarction, heart failure with HCM/COVID negative group.

**Table 2:** Clinical outcome characteristics for patients with hypertrophic cardiomyopathy comparing SARS-CoV-2 positive versus negative in baseline and propensity score matched cohorts.

Characteristics, no (%)	Unmatched				Propensity Matched			
	HCM/COVID				HCM/COVID			
		Negative	Positive			Negative	Positive	
	N	(n = 5225)	(n = 405)	*p*-Value ^1^	N	(n = 1189)	(n = 397)	*p*-Value ^1^
Death	5630	228 (4.4)	46 (11.4)	<0.01	1586	60 (5)	43 (10.8)	<0.01
Hospitalization counts	5630			<0.01	1586			<0.01
1–2		1047 (20)	172 (42.5)			273 (23)	165 (41.6)	
3–5		236 (4.5)	65 (16)			52 (4.4)	65 (16.4)	
>5		61 (1.2)	20 (4.9)			14 (1.2)	20 (5)	
Hospitalization days, median (IQR)	5630	0 (0–0)	2 (0–11)	<0.01	1586	0 (0–0)	2 (0–10)	<0.01
ICU hospitalization counts	5630			0.07	1586			0.09
1		68 (1.3)	11 (2.7)			15 (1.3)	11 (2.8)	
2		4 (0.1)	0 (0)			1 (0.1)	0 (0)	
Covid hospitalization ^2^	5630	0 (0)	141 (34.8)	<0.01		0 (0)	135 (34)	<0.01
Heart failure hospitalization ^2^	5630	427 (8.2)	100 (24.7)	<0.01	1586	104 (8.7)	96 (24.2)	<0.01
NSTEMI hospitalization	5630	175 (3.3)	35 (8.6)	<0.01	1586	55 (4.6)	34 (8.6)	<0.01
Peak hsTrop, median (IQR)	865	21 (8–62)	27 (10–136)	0.07	348	22 (8–53)	27 (9–122)	0.16
Peak BNP, median (IQR)	1139	222 (87–522)	190 (85–588.5)	0.70	410	232 (86–521)	190 (86–603)	0.76
Peak CRP, median (IQR)	688	6.4 (2.0–24.0)	38.7 (9.0–109.2)	<0.01	291	5.5 (1.8–21.3)	34.0 (9.0–106.9)	<0.01

^1^ Chi-squared, Fisher’s exact test, two sample t-test, or Wilcoxon test as appropriate. ^2^ Covid hospitalization and heart failure hospitalization are overlapped for some patients.

## Data Availability

The original contributions presented in the study are included in the article/supplementary material, further inquiries can be directed to the corresponding author/s.

## Supplementary Materials

The following supporting information can be downloaded at: https://www.mdpi.com/article/10.3390/jcdd11040104/s1. Table S1: Standardized mean differences before and after propensity-matching in SARS-CoV-2 positive vs. SARS-CoV-2 negative.
