# Supplementary material for: The Clinical Impact of SARS-CoV-2 on Hypertrophic Cardiomyopathy
Source: J Cardiovasc Dev Dis. 2024 Mar 29;11(4):104. doi: 10.3390/jcdd11040104 (PMC11050677; doi:10.3390/jcdd11040104)
Supplement: Supplementary file 1 [file jcdd-11-00104-s001.zip › jcdd-2893118-supplementary.pdf]

Supplemental table 1. Standardized mean differences before and after propensity-matching in SARS-CoV2 positive vs. SARS-CoV2 negative.

	Standardized mean differences	
**Variable**	**Unmatched**	**Propensity Matched**
Age	-0.05	0.03
BMI	0.12	-0.01
Sex	0.12	-0.01
Tobacco use	-0.05	0
Hypertension	0.33	0.04
Hyperlipidemia	0.20	0
Myocardial Injury/Infarction	0.10	-0.02
Heart Failure	0.05	0.02
